# A mechanical metamaterial with reprogrammable logical functions

**DOI:** 10.1038/s41467-021-27608-7

**Published:** 2021-12-13

**Authors:** Tie Mei, Zhiqiang Meng, Kejie Zhao, Chang Qing Chen

**Affiliations:** 1 Department of Engineering Mechanics, CNMM and AML, Tsinghua University, 100084 Beijing, PR China; 2 School of Mechanical Engineering, Purdue University, West Lafayette, IN 47907 USA

**Keywords:** Mechanical engineering, Materials science

## Abstract

Embedding mechanical logic into soft robotics, microelectromechanical systems (MEMS), and robotic materials can greatly improve their functional capacity. However, such logical functions are usually pre-programmed and can hardly be altered during in-life service, limiting their applications under varying working conditions. Here, we propose a reprogrammable mechanological metamaterial (ReMM). Logical computing is achieved by imposing sequential excitations. The system can be initialized and reprogrammed via selectively imposing and releasing the excitations. Realization of universal combinatorial logic and sequential logic (memory) is demonstrated experimentally and numerically. The fabrication scalability of the system is also discussed. We expect the ReMM can serve as a platform for constructing reusable and multifunctional mechanical systems with strong computation and information processing capability.

## Abstract (web summary)

Mechanical logic, while slower than its electronic counterpart, has the potential to be integrated into mechanical devices and a robustness in extreme environments. In this manuscript, Mei et al demonstrate reprogrammable mechanical logic which can perform combinatorial and sequential logic.

## Introduction

Mechanical computation is an old conception that probably originated from Babbage’s analytical engine in 1837^1^. Although the subsequent electronic transistor^2^ and magnetic storage^3^-based computation approach is advantageous in terms of calculation speed and data density, the robustness of mechanical logic is beneficial for applications under extreme conditions^4,5^, such as high temperature and radiation exposure, while maintaining their designed logical functions. Moreover, potential applications of mechanical computation for constructing intelligent mechanical systems have renewed the interest in mechanical computation in the last decade^6–8^. The intelligence of mechanical systems including soft robotics^9,10^, microelectromechanical systems (MEMS)^11,12^, and robotic materials^13,14^, depends upon their embedded logical functions. Such systems can sense and analyze external stimuli and, based on the results, provide appropriate responses.

Recent advances in metamaterials have facilitated the design of mechanical logic gates and the integration of them into matrix materials to develop mechanical intelligent systems. As engineered materials, metamaterials usually consist of regularly arranged building blocks (unit cells). By properly designing the building blocks and their interaction, they can have novel and even counterintuitive optical^15–17^, acoustic^18–20^, thermal^21,22^, and mechanical^23–25^ properties. The almost indefinitely available design space of metamaterials’ geometry renders it an excellent venue to explore and program new properties and functions. Mechanical metamaterials^23–25^, a subgroup of metamaterials, possessing programmable and novel deformation modes and mechanical property, are a good platform to design logical functions. A unit cell in a mechanical metamaterial possessing two different geometries can be used to represent binary states 0 and 1^26,27^. One can then realize different logic gates^28–30^ and signal transmission^31,32^ by elaborately designing the interaction between unit cells, as demonstrated in origami-based^32^ and curved-beam-based^29^ mechanical metamaterial logic systems and more advanced functional structures such as a combination lock^28^, and a “flytrap”^33^.

It is noticed that logical functions of mechanical metamaterials are usually determined by their layout and, once designed, are difficult to be altered during in-life service, which limits their applications in multi-working scenarios. Besides, when a computation is finished, the whole mechanical metamaterial system usually switches to a stable state different from its initial stable state and loses its original logical function^28,29,32,33^. Therefore, an initialization strategy is required for reusing the mechanical metamaterial system, which has yet to be fully explored. Moreover, most available mechanical metamaterial-based logic systems are not function-complete^30,32^ or cannot realize sequential logic^28,29^. As a result, they do not possess strong mechanical computation capability.

Here, a reprogrammable mechanological metamaterial (ReMM) is proposed, including a periodic array of basic logical structures with a function-complete mechanical logic gate. Under the successively imposed excitation of a series of electromagnets, the ReMM can perform different logical functions by reprogramming the loading positions determined by the on-off state’s distribution of a bistable curved beam array. Applications of the ReMM to fulfill universal combinatorial logic and sequential logic (information storage) are demonstrated. Moreover, the information storage capacity of the system is shown to be beneficial to the compact design of mechanical logic circuits. The developed ReMM is expected to provide a venue for constructing multifunctional and reusable metamaterials with strong computation power and relatively small spatial scale and thus benefit the development of mechanical systems with embedded intelligence.

## Results

### Curved-beam-based signal element and basic logical structure

**Fig. 1:**
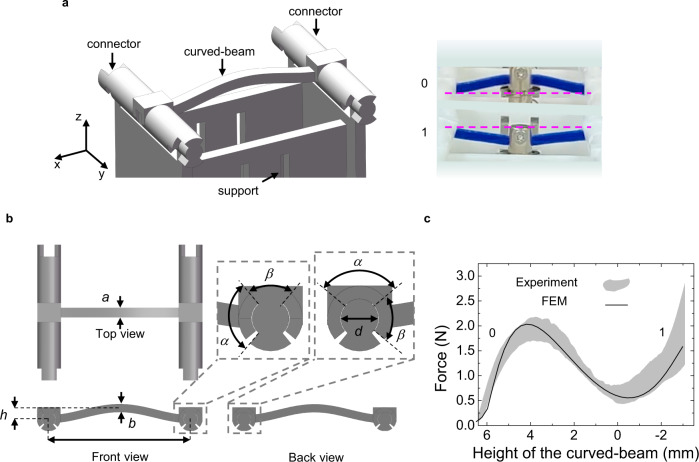
Curved-beam-based signal element. **a** A signal element consisting of two connectors, one curved beam and one support. The right insert defines the logic states of the signal element. **b** Definition of the geometrical parameters of a signal element. **c** Experimentally measured compressive force-height response of 12 normal-sized signal elements shown as the gray area and the corresponding FEM simulated result denoted by the solid line.

We will construct a curved-beam-based ReMM and demonstrate its feasibility via a combined experimental and numerical study. The mechanical property and application of curved beams have been intensively discussed^34–36^. Here, we design a special sleeve connector to connect a curved beam. A curved beam together with two connectors and a support serves as a signal element (Fig. 1a). The curved beam is made of elastic thermoplastic polyurethanes (TPU), while the connectors and support are made of photosensitive resin (DSM IMAGE8000) with an elasticity modulus much greater than that of the curved beam. The sleeve connectors can rotate around the *y* direction. Thus, the supporting boundary conditions of the curved beam can be regarded as simply supported (hinged) at its two ends. The right part of Fig. 1a shows two 3D-printed signal elements whose logic state is defined by the relative position of the midpoint of the curved beam to the horizontal line connecting the center points of the two sleeve connectors. When the midpoint is above or below the horizontal line, the logic state of the signal element is defined as 0 or 1, respectively. Figure 1b shows the geometrical parameters of the signal element. There are two sizes of the curved-beam-based signal elements, i.e., normal and small sizes. For a normal-sized signal element, *h* = 6.4 mm, *b* = 3.5 mm, *L* = 60 mm, *a* = 4 mm, *d* = 6 mm, *α* = 1.86 rad, *β* = 1.28 rad. For a small-sized signal element, *h* = 3.5 mm, *b* = 2.6 mm, *L* = 30 mm, *a* = 2.9 mm, *d* = 6 mm, *α* = 1.86 rad, *β* = 1.28 rad.

When a downward force is imposed at the midpoint of the curved beam, it deforms. The experimentally measured compressive force-associated height responses at the midpoint of 12 normal-sized signal elements are shown in Fig. 1c as the gray area. The finite element method (FEM)-based numerically simulated result is included as the solid line. Good agreement between experiment and simulation is evident. Details of the FEM modeling are given in Supplementary Note 1. The compressive responses of the small-sized signal elements are presented in Supplementary Note 2. It should be noted that the geometrical parameters of the curved beam must be carefully chosen so that it is monostable while snap-through of the curved beam under compression is allowed. As a result, the logic states 0 and 1 as marked in Fig. 1(a, c) of a loaded signal element can be easily distinguished according to whether snap-through occurs or not. Moreover, the signal element can restore to its initial state by releasing the load and a simple method for initialization can be designed.

**Fig. 2:**
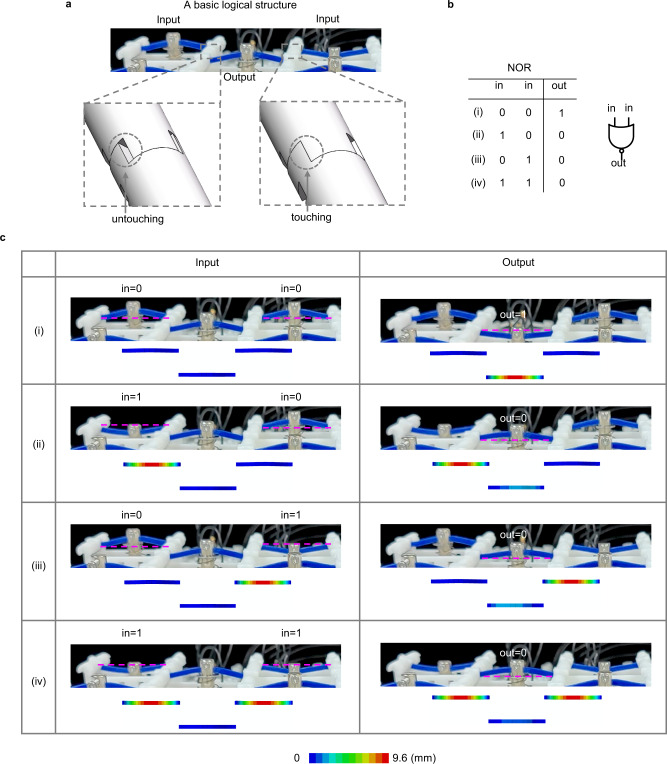
Curved-beam-based basic logical structure (NOR gate). **a** Basic logical structure comprising three signal elements, with enlarged views of the untouching and touching states of the two connectors. **b** Truth table of NOR gate. **c** Experimental and FEM simulated computation processes of the basic logical structure. The contours represent FEM calculated displacement. (i), (ii), (iii), and (iv) refer to different logic inputs indicated in **b**.

A basic logical structure is the key component to realize different logical functions in the ReMM. It comprises three curved-beam-based signal elements, i.e., two input elements and one output signal element (see, Fig. 2a). Two adjacent signal elements interact with each other via their connectors. When the two adjacent signal elements are of the same logic state, their connectors do not touch each other. When one of the signal elements switches its logic state, its connector rotates around the shaft, followed by touching and rotating the connector of the neighboring signal element. Enlarged views of the untouching and touching states of two connectors are included in Fig. 2a. The touching constraint can raise the critical snap-through force of a signal element (details are discussed in Supplementary Note 2). Thus, the logical state of two input signal elements determines the snap-through force of the output one. In a computation process, the input signal elements are loaded first. The basic logical structure outputs its computation result when excitation is applied at its output signal element. A basic logical structure with (without) the excited output signal element is called active (inactive) basic logical structure.

A NOR gate is realized when the loading force is between the two snapping forces of the signal element with and without a touching constraint. In the experiments, the signal elements are loaded by an electromagnet which pulls the curved beam down if powered on. The voltage applied to the electromagnet is so chosen that the pulling force meets the requirement to realize a NOR gate. Figure 2b shows the truth table of a NOR gate basic logical structure, with the corresponding computation processes in experiments and simulations given in Fig. 2c. For case (i), when no input curved beam is excited, the logic input of the basic logical structure is (in, in) = (0, 0). For cases (ii) and (iii), one of the input curved beams is excited, and the corresponding logic inputs are (in, in) = (1, 0) and (in, in) = (0, 1), respectively. For case (iv), however, both two input curved beams are excited, with the corresponding logic inputs being (in, in) = (1, 1). Then, the output curved beam is excited to activate output. For case (i), the connectors between adjacent beams are untouched and impose no constraint on the output curved beam before it is loaded. As a result, snap-through of the output curved beam in case (i) takes place and results in a state switch from 0 to 1. On the contrary, the state of at least one of the input curved beams of cases (ii), (iii), and (iv) differs from that of the output curved beam before it is loaded, and the connectors between adjacent beams are touched, giving rise to additional constraints to prevent snap-through of the output curved beams. Therefore, the outputs of cases (ii), (iii), and (iv) are 0. In Fig. 2c, the displacement contours corresponding to the experimentally observed deformed states are obtained by FEM, showing a good correlation between experiments and numerical simulations. The results in Fig. 2c show that the truth table of NOR gate can indeed be reproduced by the computation of the curved-beam-based basic logical structure. The computation process of the NOR gate can be found in Supplementary Movie 1.

By tuning the loading force, a basic logical structure can achieve different logical functions. In addition to NOR gate, the same basic logical structure can also serve as a NAND gate, provided that the loading force is bigger than the snapping force of a signal element with one connector imposed the touching constraint but smaller than that of a signal element with two connectors imposed the touching constraint. The realization of a NAND basic logical structure is demonstrated in Supplementary Note 2.

The basic logical structure can either serve as a functionally complete logic gate set {NOR} or {NAND} and provides a basis to fulfill universal combinatorial logic. Although including more types of logic gates may increase the computational speed of ReMM, it will render the structural design and power supply strategy much more complicated. For the sake of conceptual design with simplicity, a NOR-gate-based ReMM will be constructed. Note that it is straightforward to change the ReMM to a NAND-gate-based one by adjusting the power supply of the electromagnets, which may have improved computation efficiency of certain logical functions.

In addition, a basic logical structure with different designs is also possible. For example, a planar design is given in Supplementary Note 3. Thus, the concept of the proposed ReMM can also be extended to other systems.

### Curved-beam-based reprogrammable mechanological metamaterial

**Fig. 3:**
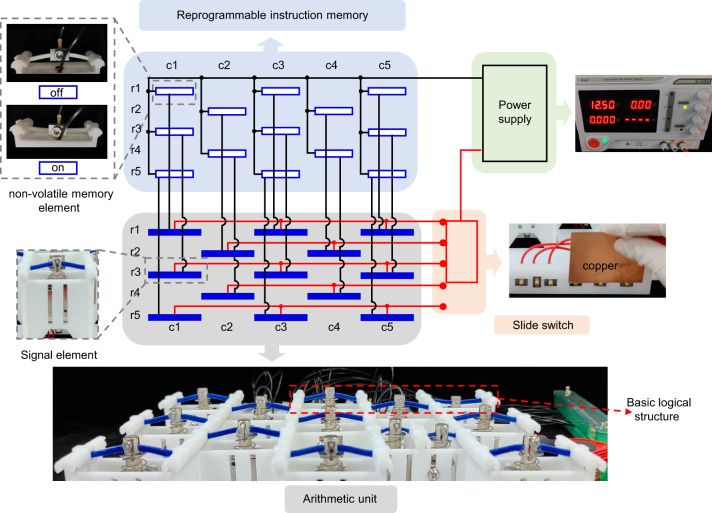
Reprogrammable mechanological metamaterial (ReMM). ReMM can be divided into four parts, i.e., the reprogrammable instruction memory, the arithmetic unit, the slide switch, and the power supply. The reprogrammable instruction memory consist of non-volatile memory elements and the arithmetic unit consists of basic logical structures.

The ReMM can be divided into four parts, i.e., an arithmetic unit (AU), a reprogrammable instruction memory (ReIM), a power supply, and a slide switch. These four parts are connected following the circuit shown in Fig. 3. The AU consists of the basic logical structures. The ReIM consists of non-volatile memory elements made of bistable curved beams (the enlarged view in Fig. 3). The non-volatile memory element can be stable in two states, i.e., the on and off states. More details of the memory element can be found in Supplementary Note 2. Turning the non-volatile memory element to the on (off) state can close (break) the corresponding circuit branch. The signal elements and non-volatile memory elements are numbered according to the row and column they locate, i.e., r*i* and c*j* meaning the *i*th row and *j*th column, respectively.

In the circuit, every signal element is connected to a non-volatile memory element in series with the same row and column number. All the signal elements with their corresponding memory elements are connected in parallel. The circuit can be divided into several branches according to the row number of the signal elements. When sliding the slide switch, these circuit branches will get closed successively and excite the signal elements row by row (from r1 to r5 or r5 to r1 in Fig. 3, depending on the sliding direction). Determined by the size of the slide switch, the signal elements of three different rows can be excited at most. Whether a signal element will be excited in a computation process is related to the state of its corresponding non-volatile memory element. Thus, the on-off state distribution of ReIM determines the combination pattern of active basic logical structures, i.e., programming the on-off state distribution of ReIM can program the instruction (logical function) executed by the AU when sliding the slide switch.

The memory element can transform between different states via elastic deformation. It is possible to change the on-off state distribution of the ReIM repeatedly. Information rewritten strategy similar to ref. ^27^, where the state of a bistable element is rechanged by electromagnetic coils, can be used to reprogram the instruction stored in ReIM. What’s more, the slide switch at its initial position does not close any circuit branches. Note that all signal elements are monostable. The AU can spontaneously be initialized by initializing the slide switch. Once initialized, the ReMM can be reused. In the following part of this paper, all different logical functions in the experiment are realized in the same ReMM system by rewriting the ReIM and initializing the AU, demonstrating the reprogrammability and reusability of the metamaterial. It should be noticed that the non-volatile memory elements can be modified to be purely mechanical so that the ReMM system is more robust against harsh environments or high temperatures. The related discussion is given in Supplementary Note 4.

### Combinatorial logic

**Fig. 4:**
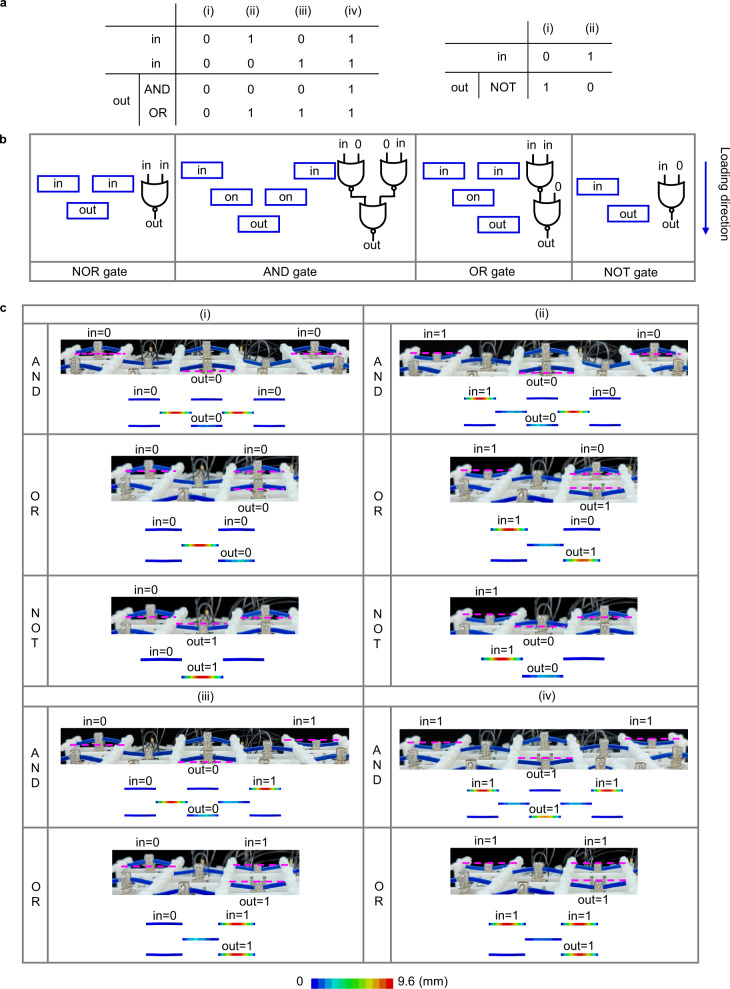
Realization of logic gates NOT, OR, and AND. **a** Truth tables of AND, OR, and NOT. **b** Distribution of the on-off states of the ReIM to realize NOR, AND, OR, and NOT gates. If the non-volatile memory element marked with “in” is in on(off) state, the corresponding input port of logic gates will receive a signal input 1(0). The non-volatile memory element marked with “out” is in on state. Other elements are in off state and are ignored. **c** Experiment realizations and the corresponding FEM displacement contours of the logic gates with different logic inputs.

The basic logical structure serves as a NOR gate, which is a functional-complete binary logic gate set and is capable of realizing universal combinatorial logic^37^. By programming the ReIM, three basic logic gates (NOT, OR, AND) can be obtained by combining NOR gates, as shown by the experiment and numerical simulation in Fig. 4. Figure 4a shows the truth tables of the three logic gates, with their on-off state distributions of ReIM and that of the NOR gate given in Fig. 4b. Note that, when sliding the slide switch, the corresponding signal elements are successively loaded following the direction indicated by the blue arrow inserted to the right of the figure. A NOT gate is obtained if one of the input signal elements of the NOR gate is not excited; if a NOT gate is activated and connected with the output port of an active NOR gate, an OR gate is realized; as for the AND gate, it is constructed by activating two NOT gates and connecting them with the input ports of an active NOR gate. The experimental and numerical realizations of the corresponding logic gates are presented in Fig. 4c. The computation procedure can be seen in Supplementary Movie 3 to Movie 5, demonstrating that the curved-beam-based ReMM successfully realizes the logical functions in Fig. 4a.

**Fig. 5:**
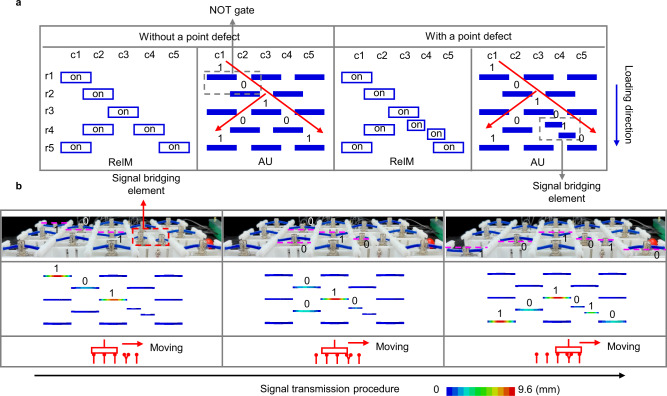
Signal transmission and bifurcation in ReMM. **a** Two examples of signal transmission and bifurcation in a perfect metamaterial and a defected metamaterial, respectively. Both the on-off state distribution of ReIM and the logical state distribution of the AU are marked. **b** The signal transmission process is shown for a defected metamaterial in experiment and FEM simulation, with the change of slide switch’s position also indicated.

To fulfill universal combinatorial logic, a functionally complete set of logic gates and a strategy for signal transmission and bifurcation are needed. In the ReMM, a signal is transmitted in a series of active NOT gates. Figure 5a shows two examples in which the signal transmits in a defective and perfect metamaterial, respectively. For the example of a perfect metamaterial, the information (logic state 0) of signal element at (r1, c1), i.e., row r1 and column c1, is transmitted to signal elements at (r3, c3), (r5, c1), and (r5, c5). Note that the signal can bifurcate because the output port of a NOT gate can serve as the input ports of more than two NOT gates. However, the logical state of adjacent elements must be different in the NOT-gate-based signal transmission paths. Thus, the signal elements in the odd-numbered rows and even-numbered rows must be in different states too, that is, information can only transmit in the odd(even)-numbered rows.

To solve the problem that information cannot transmit to any signal elements, a signal bridging element (point defect) is introduced into ReMM by replacing one normal-sized curved beam with two small-sized ones. The signal bridging element renders it possible for information transmitting between the signal elements in odd-numbered and even-numbered rows. As the example of a defective metamaterial in Fig. 5a shows, the information at (r2, c2) can transmit to the signal element (r5, c5). Figure 5b gives the corresponding experimental and FEM results. Change of the slide switch’s position is also illustrated in the third row in this figure, where the vertical red lines represent circuit branches and the ‘Y’ shaped lines refer to the circuit branches connecting the signal bridging element. The signal transmission process can be found in Supplementary Movie 6.

Note that in prior works involving buckling beams^7,29,33^ there is no way to input energy to the system along the signal transmission path, limiting the number of gates that can be cascaded until the propagating energy is not enough to trigger buckling. In our system, the signal elements along the signal path can receive energy input through successively applied excitations, which can help the information to be transmitted without attenuation over long distances. Therefore, although metamaterials contain a large number of signal elements, a signal can be transmitted to any signal element in the metamaterial by proper design of signal paths and signal bridging elements. In fact, cascading different logic gates with the mentioned signal transmission strategy, a universal combinatorial logic can be designed, if enough signal elements are included.

**Fig. 6:**
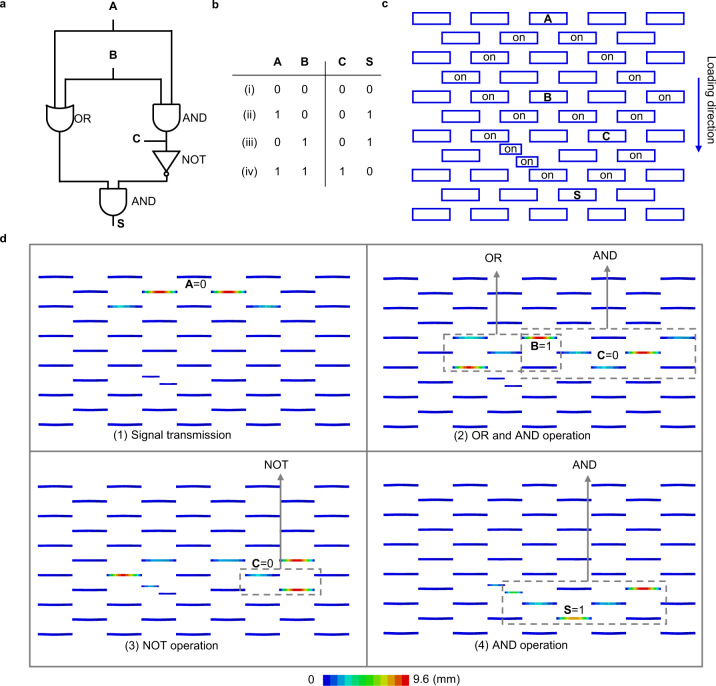
A half adder realized by ReMM. **a** Logic circuit and **b** truth table of a half adder. **c** Distribution of the On-off state of the ReIM for a half adder. **d** Four typical FEM displacement contours for the half adder for case (iii) in the computation process. Key areas served as different logical functions are indicated by dash lines.

An example of realizing universal combinatorial logic is the implementation of a half adder in Fig. 6. Figure 6(a, b) shows the corresponding logic circuit and truth table of the half adder, which calculates the sum of two single-digit numbers in binary, represented by the logic states of the input ports A and B. The computation result is represented by the logic states of two output ports S and C, with C and S being the carry digit and sum digit, respectively. The on-off state distribution of the ReIM for the half adder is given in Fig. 6c, with Fig. 6d being the FEM predicted computation process for case (iii) in Fig. 6b. Four typical FEM displacement contours illustrate how the logic gates in Fig. 6a work. The FEM simulated computation procedure to mimic the half adder’s computation for cases (i) to (iv) is shown in Supplementary Movie 7. Their computation results are summarized in Supplementary Fig. 6. These results match the truth table in Fig. 6b. Although the realization of half adder is only shown numerically, the consistency between experiments and simulations in Figs. 1, 2, 4, and 5 provide added confidence that the numerical results are adequate to show the feasibility of implementing complex logical functions via ReMM.

As another example of realizing universal combinatorial logic, a crossover (Supplementary Note 5) is numerically demonstrated. Importantly, the signal transmission paths cannot get crossed in the AU. Otherwise, information on different paths will interact and be distorted at the cross point. To solve this problem, a crossover is needed to planarize arbitrary logic circuits and benefit their realization in the ReMM.

### Sequential logic

In addition to combinatorial logic, sequential logic can also be realized via ReMM. The output of a sequential logic depends on not only the current input but also its history. The key point in building sequential logic is to provide a strategy of information storage. Thus, not all excitations are released during the computation. In this way, some signal elements maintain their logic states, and thus historical information is stored. The stored information and new input information jointly take part in subsequent computation to achieve sequential logic, which can greatly broaden the applications of the proposed ReMM.

For information storage, maintaining the excitation on one signal element is not enough. In fact, a signal element with maintained excitation will be in logic state 1 once its adjacent signal element is released (losing the touching constraint), i.e., the logic state 0 cannot be stored. Consequently, the slide switch stops at the last two circuit branches for information storage after a computation process is finished. Two adjacent signal elements of different rows in these two circuit branches can maintain their logical states despite the release of the signal elements upstream of the signal transmission direction. These two adjacent signal elements with maintained excitation are used to store information and are called volatile storage. The information in the storage will be cleared if the slide switch is initialized, thus the storage is volatile.

**Fig. 7:**
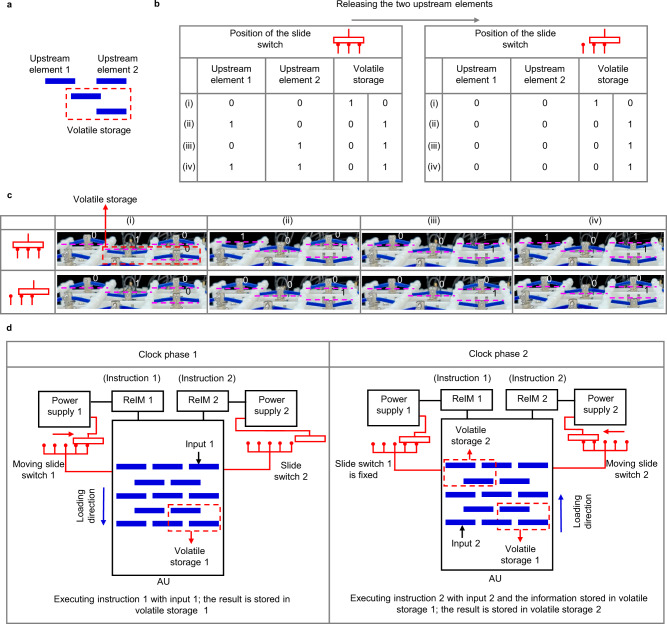
Realization of sequential logic with ReMM. **a** An illustration of a volatile storage and two signal element upstream signal transmission direction (denoted as upstream elements 1, 2). **b** logical state distribution of the volatile storage before and after the upstream elements are initialized. The corresponding experiment results are shown in **c**, where the volatile storage always maintains its logical state. **d** A typical computation process of a sequential logic. two ReIMs, powers supplies, and slide switches are used.

Here, an example is given to illustrate how the information is stored. Figure 7a shows the AU including two signal elements in the volatile storage and two upstream the signal transmission direction (denoted as upstream elements 1, 2). Figure 7b lists all possible logical state distribution of the AU before and after the upstream elements are released, with the corresponding experiment results given in Fig. 7c. The volatile storage always maintains its logic state though its adjacent signal elements are initialized. Furthermore, the stored information can also be rewritten or read with the aforementioned signal transmission strategy. The rewritten procedure is as follows: First, the volatile storage is initialized; the information can then be transmitted to the volatile storage and be stored. The read procedure is to transmit the stored information to other locations of the ReMM.

When the stored information takes part in the computation procedure, a sequential logic is realized, as shown in Fig. 7d. Two slide switches, ReIMs, and power supplies are needed for sequential logic. For clock phase 1, the slide switch moves across all the circuit branches and stops at the last two while the AU executes the instruction stored in ReIM 1 and stores the result in volatile storage 1. For clock phase 2, the stored information participates in the computation process of instruction 2 and the calculated results of instruction 2 are related to both input 2 and input 1 (historical input). Following this strategy, other complex sequential logic functions, e.g., the S-R latch, can be realized (Supplementary Note 6).

In fact, the strategy of realizing sequential logic is also beneficial to compact designs of mechanical logic circuits. In general, it is possible to divide a complex target logical function into two subfunctions which can be written into two different ReIMs. In different clock phases, subfunctions communicate with each other through the volatile storage and are executed successively to equal the target function. Consequently, a nonplanar design of logic circuits, where the AU is used twice in two clock phases, can be adopted to decrease the needed number of signal elements for a target logical function. For example, a compact design of a crossover is given in Supplementary Fig. 10. This compact crossover contains only 25 signal elements but that in Supplementary Fig. 7 contains 176 signal elements. With the developed strategy, the ReMM may serve as a platform to construct reprogrammable and reusable mechanological systems with a reduced number of needed elements.

### Scalable fabrication

Developing a scalable method for manufacturing the ReMM can broaden its application fields. The ReIM and AU are constructed using curved-beam-based elements with a modular design. These elements can be produced at different scales with different 3D printing technologies. Supplementary Figure 11 shows the 3D-printed elements at both macroscale and mesoscale. Fabrication details of the 3D-printed elements are given in Methods. With the advances in 3D manufacturing technology, it is possible to print the element at a microscale or even smaller, as shown in ref. ^29^ where a photopolymer resin was used and two-photon stereolithography and holographic optical tweezers were combined. Note that the movable joint in the elements can also be directly built in the additive manufacturing processes^38^ and eliminates the need for further assembly operations, which are difficult at the microscale.

As for the power supply and the circuit, their miniaturization strategy is well developed in the community of MEMS, e.g., arrays of micro electromagnets are constructed in ref. ^39^. Thus, considering the mechanism of the ReMM is independent of its scale, the whole system is scalable. Although such scales are still much larger than those commercially available in IC industry (e.g., 5nm-chip), they are sufficient to fulfill the application of ReMM in constructing mechanical systems with embedded intelligence^10,13,14,27,33^.

Note that the concept of the proposed ReMM can be extended to other systems (including a planar system). For example, a planar basic logical structure is constructed in Supplementary Note 3. Conventional 2D microfabrication techniques such as LIGA and silicon micromachining can be used to fabricate such planar system^6,40^. Therefore, a cheaper method to construct a micro ReMM can be obtained by improving its geometry design, i.e., redesigning a planar ReMM.

## Discussion

In summary, a ReMM capable of universal combinatorial logic and sequential logic is proposed. Its basic logical structure is multifunctional, i.e., not only function-complete logic NOR gate but also NAND gate can be realized in the same structure simply by tuning the magnitude of the applied excitations. Logical functions can be re-executed after initializing the slide switch and thus resetting the AU, and can also be reprogrammed by re-changing the on-off state distribution of the ReIM. Consider that the basic logical structure and its combination pattern to construct a target logical function are programmable. The ReMM is hierarchically reprogrammable.

Several common logic gates, signal transmission and bifurcation, and storage of information are demonstrated. A half adder, a crossover, and an S-R latch are employed to illustrate this system’s mechanical computing potential for more complex logical functions. A compact design strategy of complex logic circuits is also shown to be possible with the help of the information storage capability of ReMM. Besides, a strategy to realize purely mechanical computation is discussed which paves the way for developing mechanical computers.

The ReMM realizes complex computation functions by relating its local deformation evolution with information processing. It provides a potential approach to treat information processing as a material property and promotes the development of the unconventional computation mentioned in ref. ^41^. Accordingly, the ReMM is expected to serve as a platform for constructing reusable, multifunctional, and reprogrammable robotic material^13,14^ with robust sensing-analyzing-response function, which can benefit the development of mechanical systems with embedded intelligence. For the curved-beam-based system, the environment information can be transformed into a mechanical signal by delicately designing the beams’ material and preparation technology^33^. Then, this mechanical signal can be analyzed through the proposed mechanical computation theory and the analyzing results can get stored in the volatile storage. This stored information can further influence the global logic state distribution of the whole system via a signal transmission process. Consequently, the global mechanical property of the material is altered^27^ and is related to the analyzing results of the environment information. From this point of view, reprogrammable robotic materials with sensing-analyzing-response functions are rendered highly feasible.

## Methods

### Fabrication and testing of signal elements

The components of signal elements are designed in the CAD software Solidworks (Dassault Systèmes) and exported as STL files to be used in the subsequent 3D printing. The macroscale signal element consists of one curved beam, two sleeve connectors, and one support. The curved beam is made of thermoplastic polyurethanes (TPU) and printed using fused deposition modeling technique on an Ultimaker 3 extended printer. The sleeve connectors and support are made of photosensitive resin (DSM IMAGE8000) and printed using a Stereolithography Apparatus (SLA) UnionTech LT_450_409_G 3D printer. Then, the signal element is obtained by gluing one curved beam and two sleeve connectors together and assembling with the support We have fabricated 14 macroscale signal elements including 12 normal-sized ones and 2 small-sized ones, as can be seen in the AU in Fig. 3. In the ReMM, these elements are excited by the electromagnet KK-1039B made by Kakcom. The compressive tests of all signal elements are conducted using a uniaxial testing machine (Zwick Z005) equipped with a 50 N load cell. A 3D-printed test fixture is used in these tests to ensure that the two ends of a signal element are simply supported. Note that petroleum jelly is applied to the inner wall of the sleeve connectors to minimize friction. The mesoscale signal element consists of a curved beam with two connectors at its ends and one support. These components are all made of an ultra-tough-low-viscosity (UTL) resin and printed by projection micro stereolithography with the 3D printer microArch P150 (Boston Micro Fabrication).

### Finite elements analysis

The FEM results under the quasi-static loading are obtained using commercial finite element software ABAQUS. The explicit dynamic analysis module is selected for all simulations, except for those in Fig. 1c and Supplementary Fig. 2(a, b) in which the static general analysis module is used. Geometric nonlinearity is included in the model to simulate the large deformation behavior more precisely. The signal element is modeled by Timoshenko beam elements (B31). One signal element is meshed with 11 beam elements. Poisson’s ratio of the curved beam is set as 0.45 while TPU is nearly incompressible. The density is set as 1.2 × 10^3^kg·m^−3^ while it is not an important parameter for quasi-static loading. The elastic modulus is extracted to be 72 MPa so that the FEM results agree closely with the average results of the experiment in Fig. 1c. The viscosity of material is also considered to dissipate the kinetic energy of the system. The adopted damping coefficients are *α* = 8 × 10^−4^ and *β* = 8 × 10^−5^. Thus, the kinetic energy is very small compared to the strain energy at the time before snapping through. The modeling details of the signal element and the whole curved-beam-based system can be found in Supplementary Note 1.

## Supplementary information

Supplementary Information

Description of Additional Supplementary Files

Supplementary Movie 1

Supplementary Movie 2

Supplementary Movie 3

Supplementary Movie 4

Supplementary Movie 5

Supplementary Movie 6

Supplementary Movie 7

Supplementary Movie 8

Supplementary Movie 9

## Data Availability

The data that support the plots within this paper and other findings of this study are available from the corresponding author upon request.
