# Supplementary material for: A mechanical metamaterial with reprogrammable logical functions
Source: Nat Commun. 2021 Dec 13;12:7234. doi: 10.1038/s41467-021-27608-7 (PMC8668933; doi:10.1038/s41467-021-27608-7)
Supplement: Supplementary file 1 — Supplementary Information [file 41467_2021_27608_MOESM1_ESM.pdf]

Supplementary Materials for

A mechanical metamaterial with reprogrammable logical functions

Tie Mei, Zhiqiang Meng, Kejie Zhao, Chang Qing Chen *

*Corresponding author. Email: chencq@tsinghua.edu.cn (C.Q.C.)

Supplementary Note 1: Details of FEM modeling

Supplementary Fig. 1 shows the modeling details in the FEM simulation. Considering the elastic modulus of the connectors is much larger than that of the curved-beam, the elastic modulus of the green area in Supplementary Fig. 1a is simply set as 100 times larger than that of the blue area. The length of the green area is just the distance between the central point of the sleeve connector to the end of the curved-beam. Thus, the green area can be approximately equivalent to the sleeve connector in the simulation.

The radius of the inner wall of the sleeve connector is slightly larger than that of the cylindrical bulge on the fixture in the experiment so that the cylindrical bulge can be fitted into the sleeve connector. The difference between these radii (denoted by Δ_{gap} =0.2mm) is considered in the FEM modeling. There are two nodes at the position of the end of a signal element: one green node denoting the boundary node of the beam element at the end; one black node fixed in the space. These two nodes are connected by a nonlinear extension spring and their interaction is shown in Supplementary Fig. 1b where Δ is the distance of the two nodes. If $\Delta < \Delta_{\text{gap}}$, the stiffness of the spring is about zero. This means the inner wall of the sleeve connector does not touch the cylindrical bulge. If $\Delta \geq \Delta_{\text{gap}}$, the stiffness of the spring is set as $K = 100F_{\text{snap}} \cdot \Delta_{\text{gap}}^{-1}$ where F_{snap} is the snap-through force shown in Fig. 1c. Note that K is big enough to limit further displacement of the sleeve connector, meaning the inner wall of the sleeve connector has touched the cylindrical bulge. Moreover, there is a linear spring in the electromagnet to help its columnar iron core restore its initial position when it is power-off. This spring,

with stiffness being 2N/m, is equivalent to that between the midpoint of the beam and the ground in FE simulation.

As for the FEM simulation of the AU, the interaction between different signal elements should be considered. For this purpose, a nonlinear torsion spring pointing to z-direction is used to connect the ends of two adjacent signal elements as shown in Supplementary Fig. 1c. The torsional response of this spring is given in Supplementary Fig. 1d where $\Delta\theta$ is the relative rotational angle of the two ends of adjacent signal elements, with the definitions of α and β given in Fig. 1b. When $\Delta\theta < \alpha - \beta$, the stiffness of the rotational spring is about 0, meaning the adjacent connectors do not touch each other as shown in the left enlarged view in Fig. 2a. When $\Delta\theta \geq \alpha - \beta$, the tangent stiffness of the rotational spring is $K_\theta = 25/3Eab^3L^{-1}$ where *a*, *b*, and *L* are the width, thickness, and length of the curved beam, respectively. Note that K_θ is 100 times bigger than the bending stiffness of the curved-beam $1/12Eab^3L^{-1}$ and is big enough to represent the touching of two adjacent connectors given in the right enlarged view of Fig. 2a. During the computation process of the ReMM, the excitation provided by the electromagnet is replaced by a constant force. The FEM model is sufficient to reproduce the experiment results as shown in Figs. 2, 4, and 5.

Supplementary Note 2: Snap-through responses of curved beams and mechanism of a basic logical structure

The load-height responses for the small-sized curved beams in signal elements are summarized in Supplementary Fig. 2a. Both experiments and FEM prediction are included, agreeing well with each other. Supplementary Fig. 2b show the FEM predicted responses of a small- and a normal- sized curved beams with the springs between electromagnet and beam included. It can be seen from Supplementary Fig. 2 a and b that, although snap-through is present in all responsive curves, they are monostable and can restore its initial state (logic state 0) when external excitation is released. This monostable feature is required to initialize the AU while assure the reusability of ReMM.

As for the curved beams in the non-volatile memory elements, their responsive curves are given in Supplementary Fig. 2c. As can be seen, the force can be smaller than 0, a feature of bistability that different from those of the curved beams in signal elements (Supplementary Fig. 2a). These curved beams can be stable at the on and off states and are used for non-volatile information storage. Note that the length of the beam is 62 mm after fabrication and the beam is assembled to the support whose two ends are 60 mm apart. Thus, the beam will be buckled. The on (off) state implies that the beam be buckled upward (downward).

The interaction between adjacent signal elements has also been simulated (Supplementary Fig. 2d). The black curve shows the compressive response of a signal element in the first loading step in which the snap-through force is about 2.5 N (the same as that in Supplementary Fig. 2b). However, the snap-through force of the red line for the second loading step is increased to 3.4 N, due to the touching constraint shown in the right enlarged view of Fig. 2a. To realize a NOR gate, the loading force is set as 3 N (a slightly larger than the snap through force of a single signal element without the touching constraint but smaller than that with the touching constraint). Following such a design, once an input signal element being state 1, the output signal element cannot snap through and the truth table of NOR gate is reproduced (see, Fig. 2c).

The basic logical structure can also serve as a NAND gate if the loading force is larger. Two typical cases are used to demonstrate the implementation of a NAND gate (Supplementary Fig. 3a). In the first loading step, the signal elements are loaded with a force of 5 N in magnitude and the compressive response is shown as the black curve in Supplementary Fig. 3b and c. In the second loading step, the snap-through force of the signal element with one adjacent signal element being state 1 is about 3.8 N (red line in Supplementary Fig. 3b) while that with two adjacent signal elements being state 1 is about 8 N (blue line in Supplementary Fig. 3c). Thus, the output signal element subject to the load (5 N in magnitude) does not snap through only when there are two input elements being state 1. This procedure shows that a NAND gate is realized. Supplementary Fig. 3(d, e) gives the truth table of the NAND gate and the corresponding FEM simulation results, respectively.

Supplementary Note 3: A basic logical structure with different design

In Fig. 2, we have shown a concept to realize NOR gate. Here, we will show that the concept can be extended to other structures. Note that three requirements must be satisfied to design a different basic logical structure. First, a signal element can be used to represent two different logic states 0 and 1 via deformation. Besides, the initial state of the signal element should be the logic state 0. Second, a signal element of state 0 does not influence the state changing process of its adjacent signal elements. Third, a signal element of state 1 can hinder the state changing of its adjacent signal elements. Following these requirements, a new basic logical structure is designed, shown in Supplementary Fig. 4.

As Supplementary Fig. 4a shows, a curved beam (the same curved beam as that in the main text) is selected to construct the signal element and the definition of the logic state is also the same as that mentioned in the main text. As the left two signal elements being in state 0 show, adjacent signal elements are connected by zigzag trusses in which no axial force exists in two adjacent beams. Thus, there is no interaction between these two adjacent elements. If a signal element is switched to state 1 (the right signal element), some zigzag trusses will be straightened. The axial force in the straightened truss will rotate the end of the adjacent curved beam of which the critical force for snapping through is increased. In the design, all three requirements are satisfied.

Supplementary Fig. 4b presents the FEM simulated computation process of the basic logical structure, with the loading force being 3 N. The truth table of NOR gate is successfully reproduced, showing that the concept of ReMM can indeed be extended to other systems. Note that the newly designed basic logical structure is a monolithic planar architecture. Conventional microfabrication techniques, such as LIGA, and silicon micromachining, can be used to fabricate such structures. Thus, a cheaper method (comparing with the mentioned 3D printing method in the main text) for the microfabrication of ReMM is possible.

Supplementary Note 4: A purely mechanical computation system

As Supplementary Fig. 5 shows, a purely mechanical computation system is designed, based on the same conception introduced in the main text. A “super” element is constructed by assembling a signal element, a non-volatile memory element, and a loading element together (Supplementary Fig. 5a). Note that the bi-stable beam is horizontally placed in the non-volatile memory element and it exposes (covers) the hole below at the on (off) state. Supplementary Fig. 5b gives an illustration of the purely mechanical computation system consisting of the “super” elements and a loading system. As the rollers in the loading system move forward, the “super” elements are pressed under the belts row by row with the help of the gears to derive the computation process (Supplementary Movie 2). Supplementary Fig. 5c shows that the on-off state of the non-volatile memory element determines whether the loading bar will be blocked, i.e., whether the signal element will be activated. Thus, the logical function executed by the computation system can be programmed by changing the on-off state distribution of the non-volatile memory elements. Supplementary Fig. 5d shows the computation results of a NOR gate experimentally, demonstrating the feasibility of the proposed strategy to construct a purely mechanical computation system.

In general, the purely mechanical computation system is constructed by modifying the non-volatile memory to be purely mechanical and adding a mechanical loading system. The scenario given above is just one possible scheme out of many choices. The proposed design is expected to benefit the construction of a purely mechanical computer. (The authors would like to thank the anonymous reviewer for the suggestion of a possible purely mechanical computer system).

Supplementary Note 5: Design of a crossover

The crossover is equivalent to two crossed signal paths (Supplementary Fig. 7a) and can be achieved by cascading a series of NOR, as follows: a NAND gate can be obtained by assembling four NOR gates; four NAND gates can realize an XOR gate; a crossover can be realized by combining three XOR gates. Supplementary Fig. 7(c, d) presents the truth table of a crossover and the corresponding computation process for case (iv) represented by 8 typical steps (1) to (8). As for other cases, the computation processes can be found in Supplementary Movie 8.

Supplementary Note 6: Design of an S-R latch

Supplementary Fig. 8a shows the logic circuit of an S-R latch consisting of two parts, i.e., two NOR gates and one crossover feedback. In the ReMM, the computation process has four clock phases as summarized in Supplementary Fig. 8b in conjunction with its truth table, where Q represents the stored information, $\bar{\mathrm{Q}} = \mathrm{NOT}(\mathrm{Q})$, and S and R are the "set" and "reset" inputs. If S=1 and R=0, Q will be rewritten as 1; if S=0 and R=1, Q will be written as 0; if S=R=0, Q will remain its current logic state; if S=R=1, both $\bar{\mathrm{Q}}$ and Q will be 0 ($\bar{\mathrm{Q}}$ is not equal to $\mathrm{NOT}(\mathrm{Q})$, this condition is not allowed in the usage of an S-R latch). Details of the computation process for case (ii) are illustrated in Supplementary Fig. 9 in terms of FEM predicted displacement contours, where the volatile storages are used for the communication of logical functions in different clock phases.

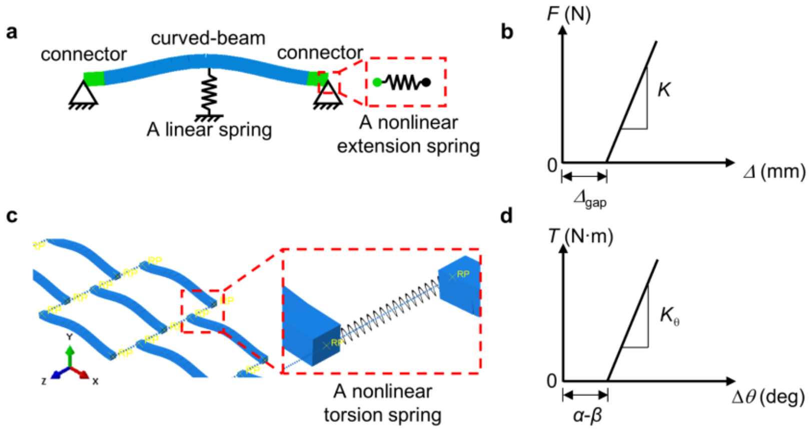

Supplementary Figure 1. Details of FEM modeling. a FEM Model of the signal element. There are three springs: two nonlinear extension springs between the end nodes (the green node) of the beam and the fixed support node (the black node) and one linear spring between the midpoint of the beam and the ground. **b** The constitutive force-displacement curve of the nonlinear extension spring. **c** FEM model of the curved-beam system. The interaction between two signal elements is modelled by a nonlinear torsion spring. **d** The constitutive torque-rotation curve of the nonlinear torsion spring.

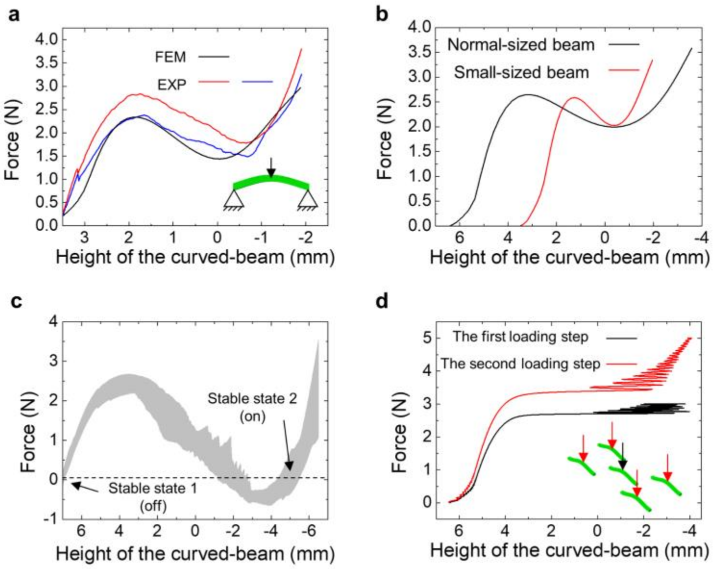

Supplementary Figure 2. Midpoint force-hight responsive curves of curved-beams. a Experimentally measured and FEM predicted responses of the small-sized curved-beams. The inset shows the imposed loading condition. Only two small-sized curved beams shown in Fig. 3 are tested. **b** FEM predicted responses of the small-sized and normal-sized curved-beams with a linear spring added between their midpoints and the ground. **c** Measured responses of the curved-beams in 14 non-volatile memory elements, two stable states (on and off) can be found in all these curves. **d** FEM predicted response of a curved-beam (black curve) and that of its four adjacent curved beams under subsequent loading (red curve).

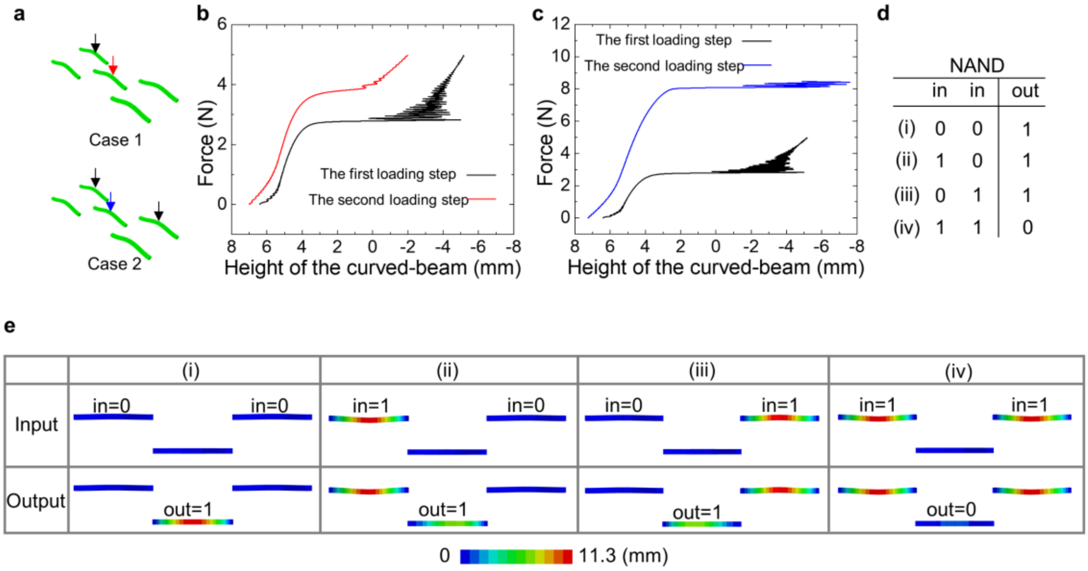

Supplementary Figure 3. The basic logical structure serving as NAND gate. a Two typical loading cases during the computation process. The force indicated by the black (red and blue) arrow(s) is applied firstly(secondly). **b** FEM predicted force-height response of a firstly loaded curved-beam and a later loaded adjacent curved beam in case 1. **c** FEM predicted force-height response of two firstly loaded curved-beams and a later loaded adjacent curved beam in case 2. **d** Truth table of NAND gate. **e** Computation process of the NAND basic logical structure represented by FEM calculated displacement contours. (i), (ii), (iii), and (iv) refer to different logic inputs indicated in **d**.

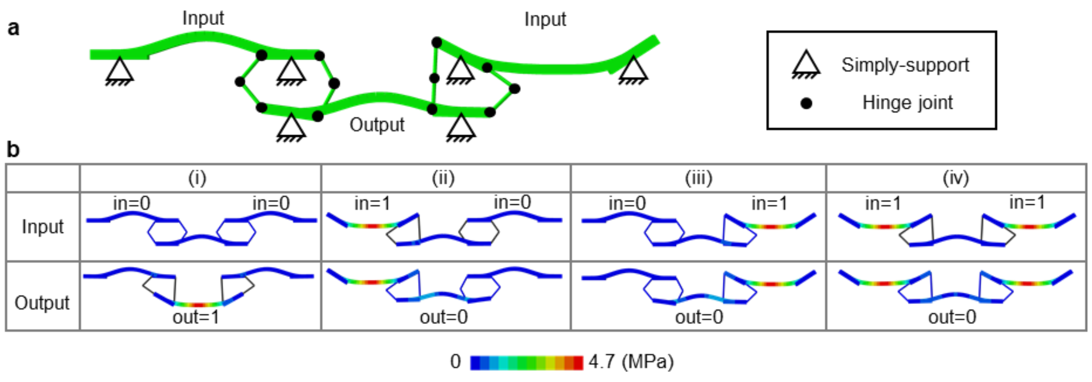

Supplementary Figure 4. A basic logical structure of different design. **a** The boundary condition of the basic logical structure and the connection between different signal elements. **b** The computation process of a NOR basic logical structure represented by some typical FEM Mises-stress contours. (i), (ii), (iii), and (iv) refer to different logic inputs indicated in Fig.2b.

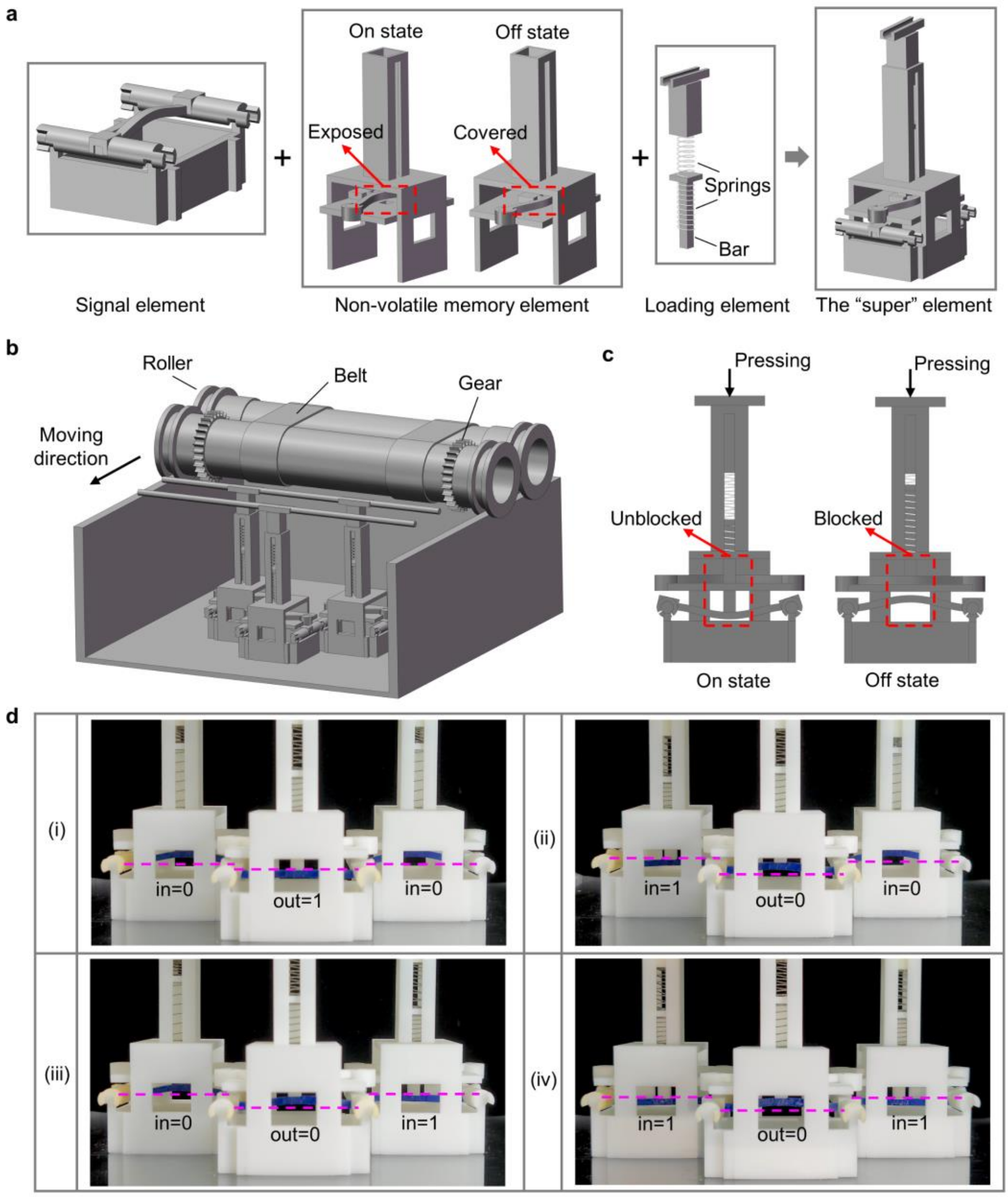

Supplementary Figure 5. A purely mechanical computation system. **a** The “super” element consisting of a signal element, a non-volatile memory element, and a loading element. **b** The mechanical computation system constructed by the “super” elements and a loading system. **c** The pressed “super” elements with different on-off states of their inner non-volatile memory element. **d** Experiment realization of a purely mechanical NOR gate with different logic inputs.

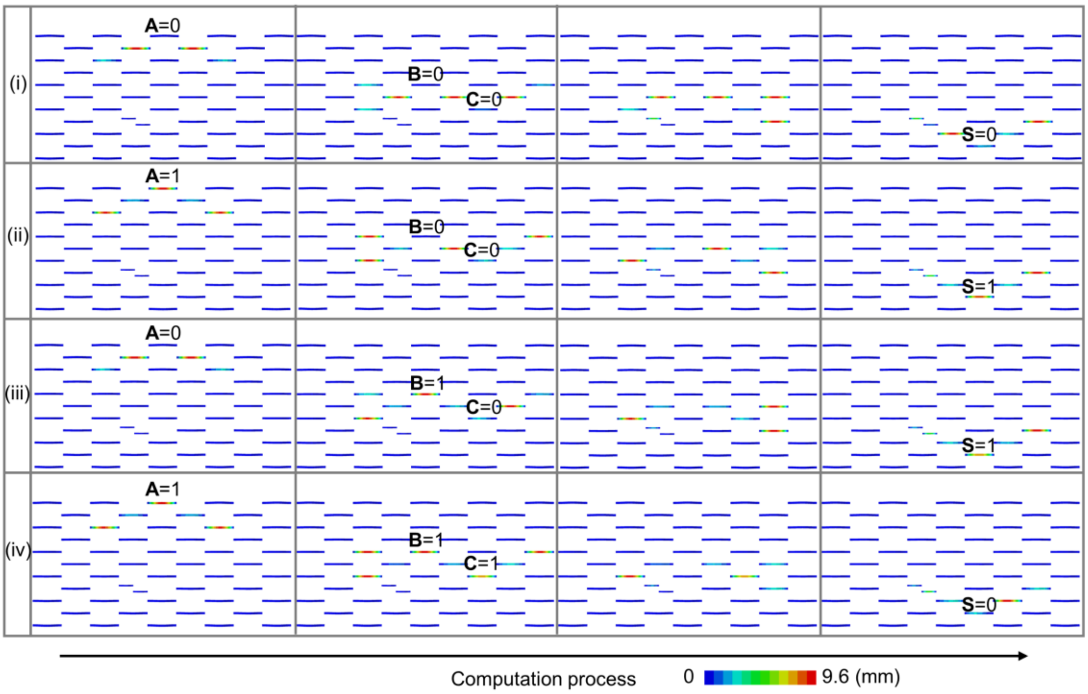

Supplementary Figure 6. FEM predicted computation process of a half adder in the ReMM for different logical inputs (i) to (iv) shown in Fig. 6b.

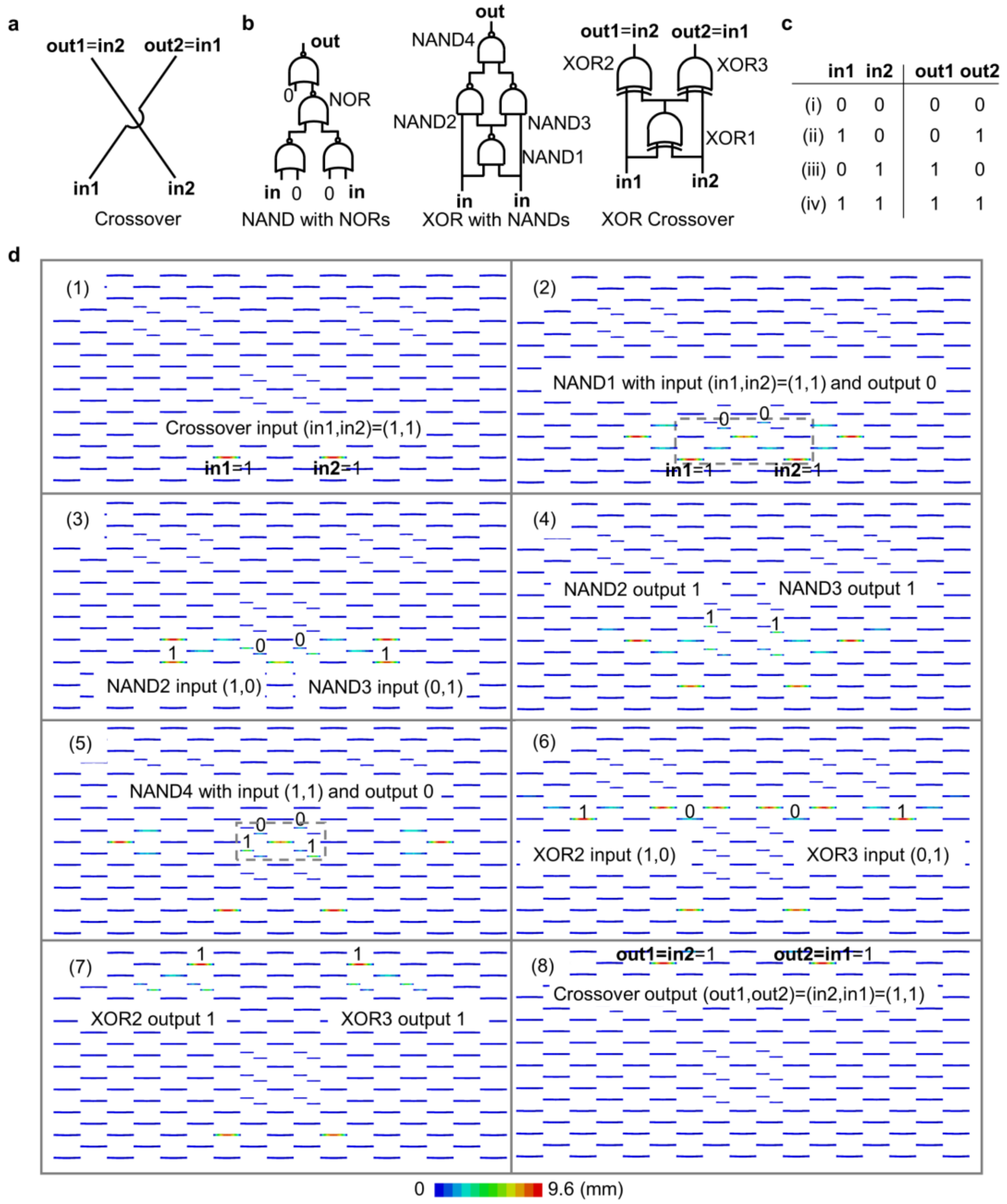

Supplementary Figure 7. A crossover. **a** Two crossed signal transmission path. **b** Construction of a crossover with NOR gates. **c** Truth table of a crossover. **d** Eight typical steps (from (1) to (8)) to show the computation process of a crossover for case (iv). Steps (2) to (5) shows the procedure of a NAND gate based XOR gate. Computation processes of the other two XOR gates needed to realize a cross are shown in (6) and (7).

a

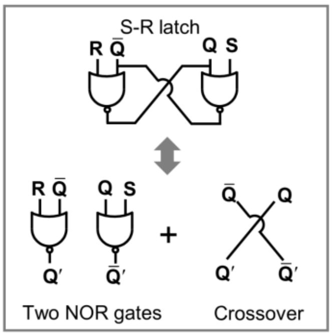

b

	Clock phase 1						Clock phase 3							
	Two NOR gates						Two NOR gates							
	input				output		input				output			
	Q	$\bar{Q}$	S	R	Q'	$\bar{Q}'$	Q	$\bar{Q}$	S	R	Q'	$\bar{Q}'$	Q	$\bar{Q}$
(i)	0	1	0	0	0	1	0	1	0	0	0	1	0	1
(ii)	0	1	1	0	0	0	0	0	1	0	1	0	1	0
(iii)	0	1	0	1	0	1	0	1	0	1	0	1	0	1
(iv)	1	0	0	0	1	0	1	0	0	0	1	0	1	0
(v)	1	0	0	1	0	0	0	0	0	1	0	1	0	1
(vi)	1	0	1	0	1	0	1	0	1	0	1	0	1	0
(vii)	1	0	1	1	0	0	0	0	1	1	0	0	0	0
(viii)	0	1	1	1	0	0	0	0	1	1	0	0	0	0
					input		output				input		output	
					Crossover						Crossover			
					Clock phase 2						Clock phase 4			

Supplementary Figure 8. Construction of a S-R latch. a Logical circuit of an S-R latch. It can be divided into two NOR gates and a crossover. **b** Truth table of the S-R latch. The computation process is also shown as four clock phases where two NOR and crossover operations are conducted twice.

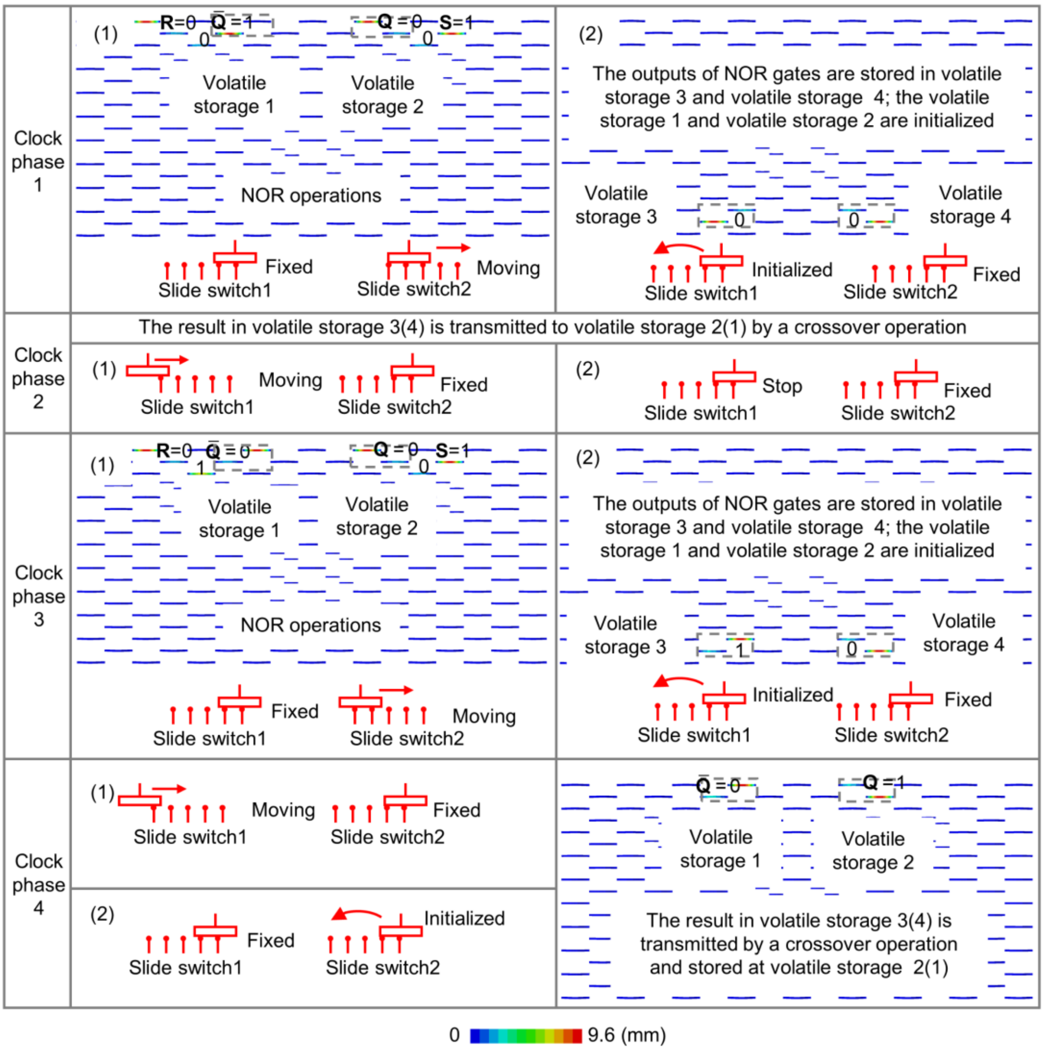

Supplementary Figure 9. Computation process of an S-R latch for case (ii). Four clock phases are indicated. In clock phases 1 and 3, the FEM displacement contours are shown for the computation process of the two NOR gates. In clock phases 2 and 4, the computation processes of the crossover are illustrated. The FEM contours of a crossover can be found in Supplementary Figure 6d. The change of the two slide switches' position during computation is also included. The slide switch 1 is related to the volatile storages 1 and 2 and the crossover instruction. The switch 2 is related to the volatile storages 3 and 4 and the two NOR instruction.

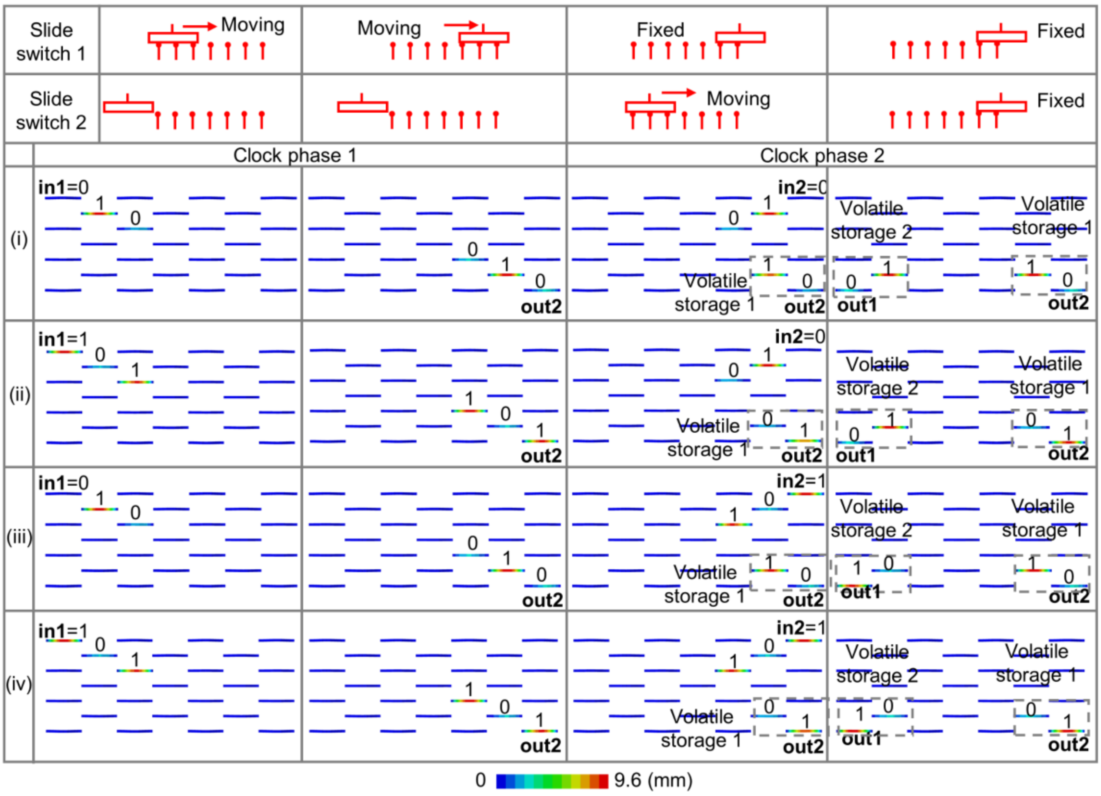

Supplementary Figure 10. Computation process of a compact crossover. FEM predicted displacement contours of the computation process for cases (i) to (iv) shown in supplementary Figure 6c. In clock phase 1(2), the information of in1(in2) is transmitted to out2(out1) and is stored at volatile storage 1(2). The slide switch 1(2) is related to volatile storage 1(2). The corresponding changes of the position of these two slide switches are also included.

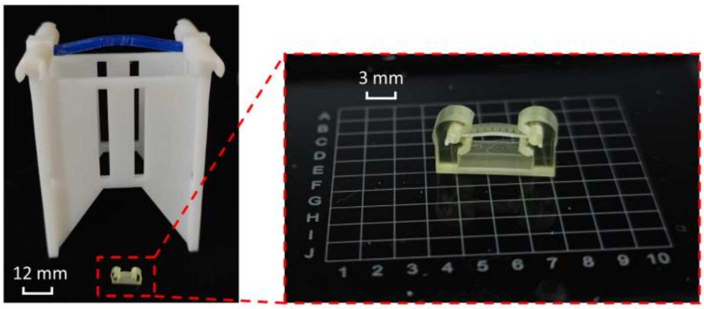

Supplementary Figure 11. Signal elements at macroscale and mesoscale. The right inset is an enlarged view of the signal element at mesoscale.
