# Supplementary material for: A mechanical metamaterial with reprogrammable logical functions
Source: Nat Commun. 2021 Dec 13;12:7234. doi: 10.1038/s41467-021-27608-7 (PMC8668933; doi:10.1038/s41467-021-27608-7)
Supplement: Supplementary file 2 — Description of Additional Supplementary Files [file 41467_2021_27608_MOESM2_ESM.pdf]

Description of Additional Supplementary Files

File Name: Supplementary Movie 1
Description: Computation process of a NOR gate in the curved-beam-based ReMM.

File Name: Supplementary Movie 2
Description: Computation process of a purely mechanical NOR gate in the curved-beam-based ReMM.

File Name: Supplementary Movie 3
Description: Computation process of a AND gate in the curved-beam-based ReMM.

File Name: Supplementary Movie 4
Description: Computation process of an OR gate in the curved-beam-based ReMM.

File Name: Supplementary Movie 5
Description: Computation process of a NOT gate in the curved-beam-based ReMM.

File Name: Supplementary Movie 6
Description: Signal transmission and bifurcation in the curved-beam-based ReMM.

File Name: Supplementary Movie 7
Description: Computation process of a half adder in the curved-beam-based ReMM.

File Name: Supplementary Movie 8
Description: Computation process of a crossover in the curved-beam-based ReMM.

File Name: Supplementary Movie 9
Description: Computation process of a compact crossover in the curved-beam-based ReMM.
